# Supplementary material for: LHP1-mediated epigenetic buffering of subgenome diversity and defense responses confers genome plasticity and adaptability in allopolyploid wheat
Source: Nat Commun. 2023 Nov 20;14:7538. doi: 10.1038/s41467-023-43178-2 (PMC10661560; doi:10.1038/s41467-023-43178-2)
Supplement: Supplementary file 1 — Supplementary Information [file 41467_2023_43178_MOESM1_ESM.pdf]

Supplemental Figures for

LHP1-mediated epigenetic buffering of subgenome diversity and defense responses confers genome plasticity and adaptability in allopolyploid wheat

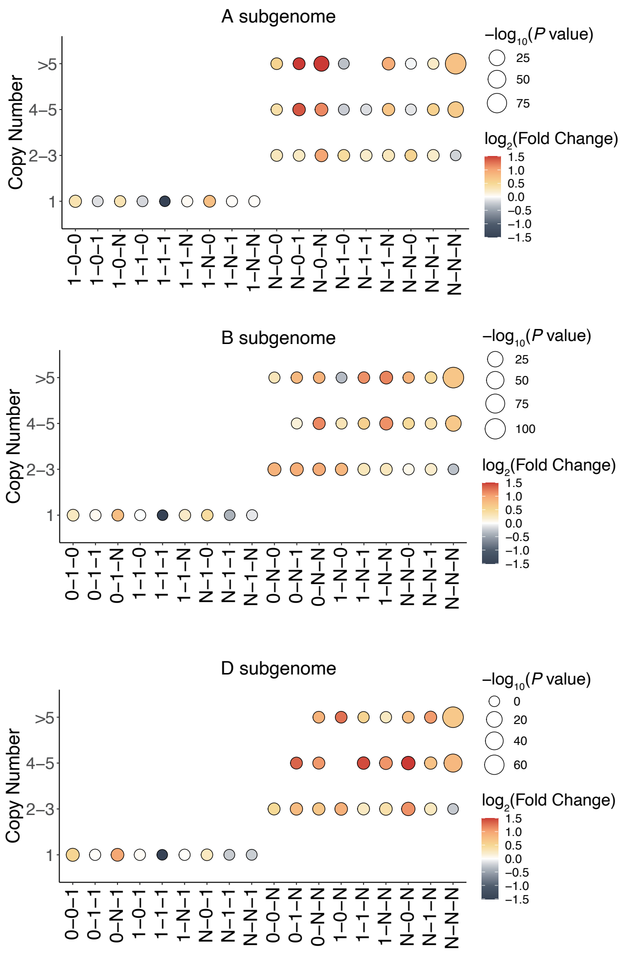

Supplemental Figure 1. Enrichment of LHP1 targeted genes in sc-triads and non-sc-homoeologs with different copy number. sc-triads and non-sc-homoeologs were divided in 4 groups in each subgenome: copy number = 1, 2 ≤ copy number ≤ 3, 4 ≤ copy number ≤ 5, copy number > 5.

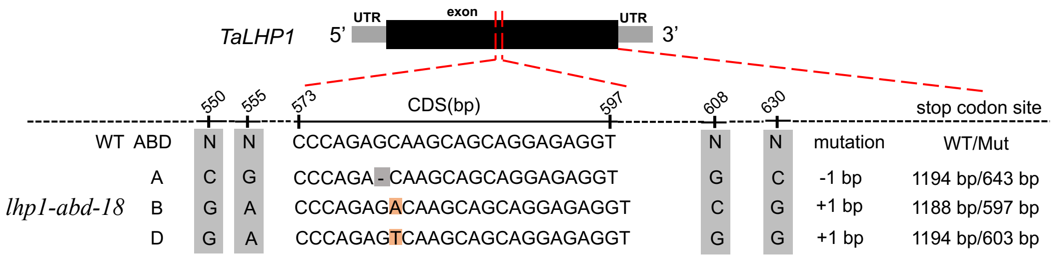

Supplemental Figure 2. CRISPR-editing sites in *Talhp1-abd-18* lines. The positions in gray represent the reference SNVs differentiating homoeologs. The region from 573 to 597 is the CRISPR target site. The type of mutation is listed on the right.

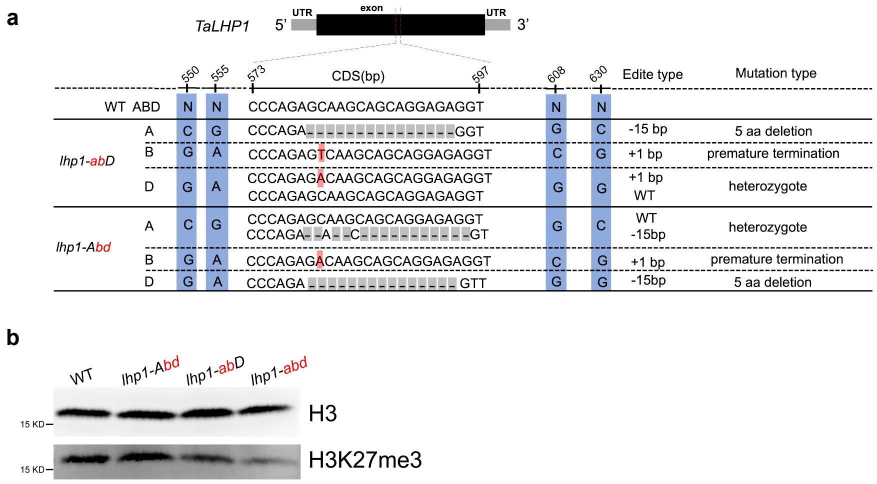

Supplemental Figure 3. Mutation information of single and double mutants and comparation of H3K27me3 level in single, double and triple mutants.

a) CRISPR-editing sites in *Talhp1-abD and Talhp1-Abd* lines. The positions in blue represent the reference SNVs differentiating homoeologs. The region from 573 to 597 is the CRISPR target site. The type of mutation is listed on the right.

b) The global H3K27me3 level in *Talhp1* triple, single and double mutants via western blot with specific antibody against H3K27me3.

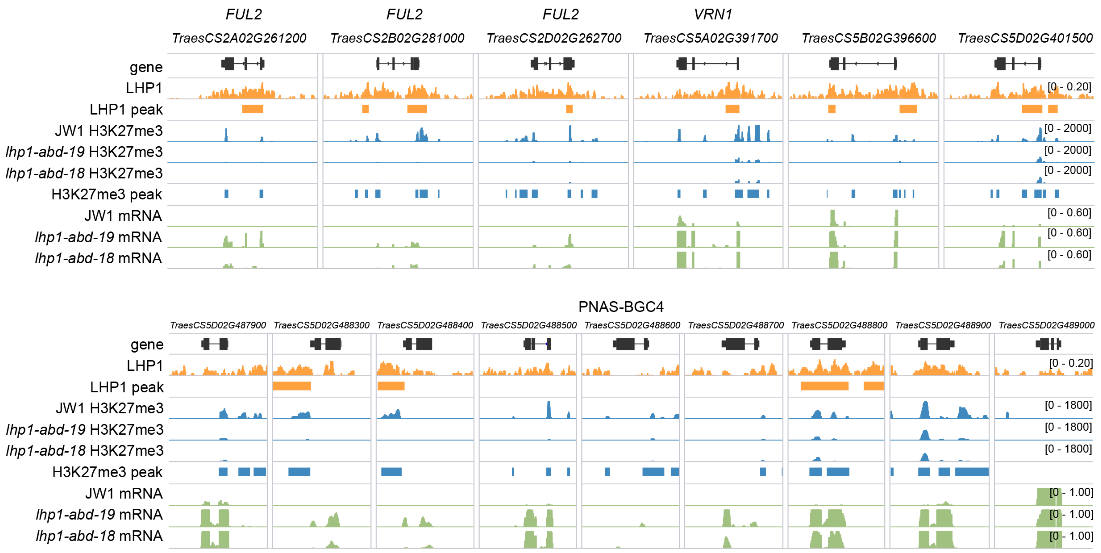

Supplemental Figure 4. Genome tracks illustrating the functional genes involved in flowering and floral development (top panel), gene clusters synthesizing defense-related metabolites (bottom panel).

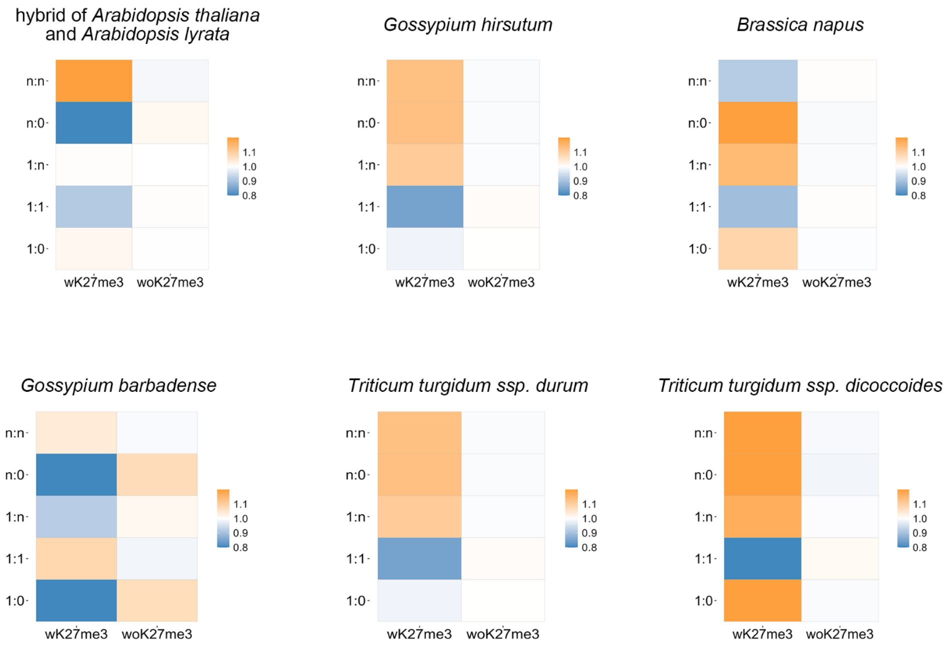

Supplemental Figure 5. H3K27me3 preferentially targeted non 1:1 homoeologous genes in polyploidy species.

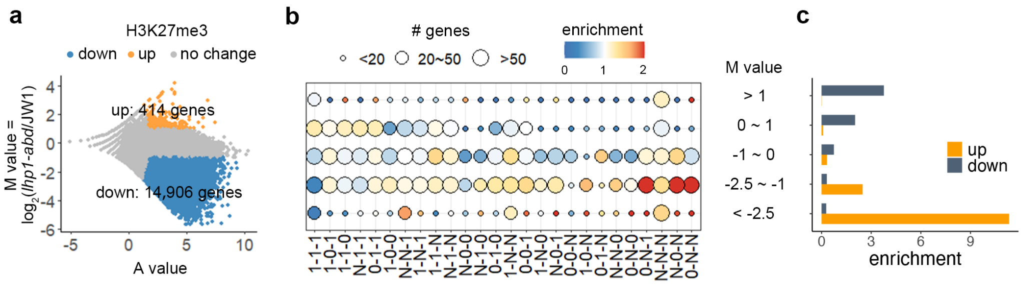

Supplemental Figure 6. Changes of H3K27me3 and expression in *Talhp1-abd-18*.

a) H3K27me3 changes in the *Talhp1-abd-18* mutant. The x-axis represents the average read densities in H3K27me3 target genes in the wild-type and *Talhp1-abd-18* samples, whereas the y-axis represents the log2 changes (*M* value) of H3K27me3 in *Talhp1-abd-18*.

b) Enrichment of homeologous groups in genes with differential H3K27me3 changes (*M* value) in *Talhp1-abd-18*. The color of dots represents enrichment score, with all high confident genes as background.

c) Enrichment of genes with up- and down-regulated expression in genes with different levels of H3K27me3 changes [represented by M = $\log_2$(fold-change)], with all high confident genes as background.

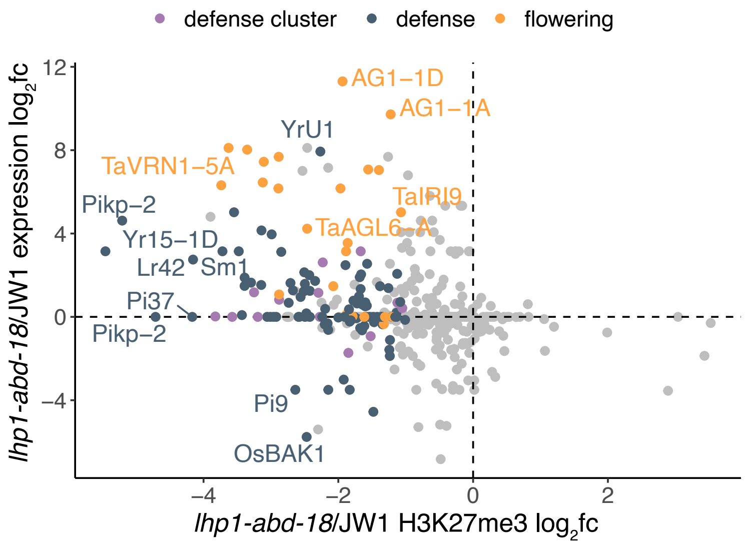

Supplemental Figure 7. Scatter plot of the changes in the expression and H3K27me3 of functional genes in *Talhp1-abd-18*. Different colors and shapes represent different functions.

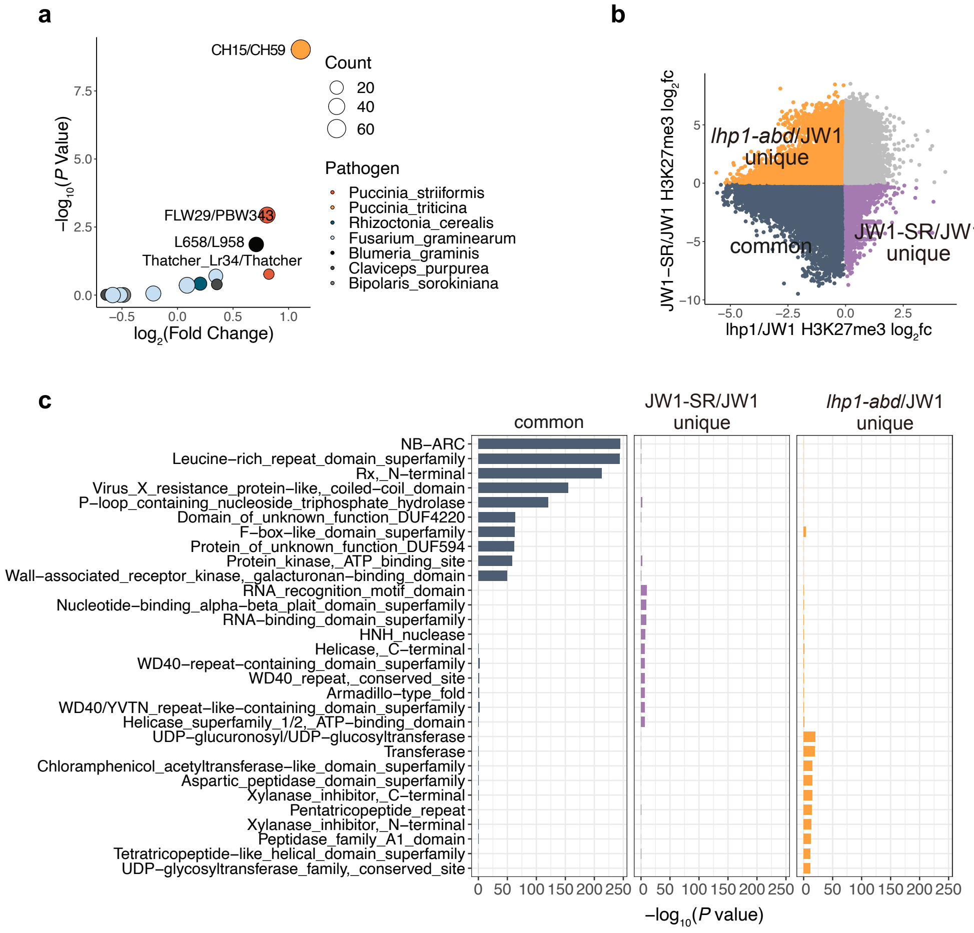

Supplemental Figure 8. Stripe rust infection induces subgenome-diversified defense genes by removing H3K27me3 (Results of *Talhp1-abd-18*).

a) Enrichment of *Talhp1-abd*-induced genes among the genes more highly expressed in samples resistant to various pathogens (represented by different colors) than in the corresponding susceptible samples.

b) Scatter plot representing H3K27me3 changes post-inoculation and in the *Talhp1-abd-18* mutant.

c) Enriched functional domains among the genes with H3K27me3 levels commonly or uniquely decreased in *Talhp1-abd-18*.

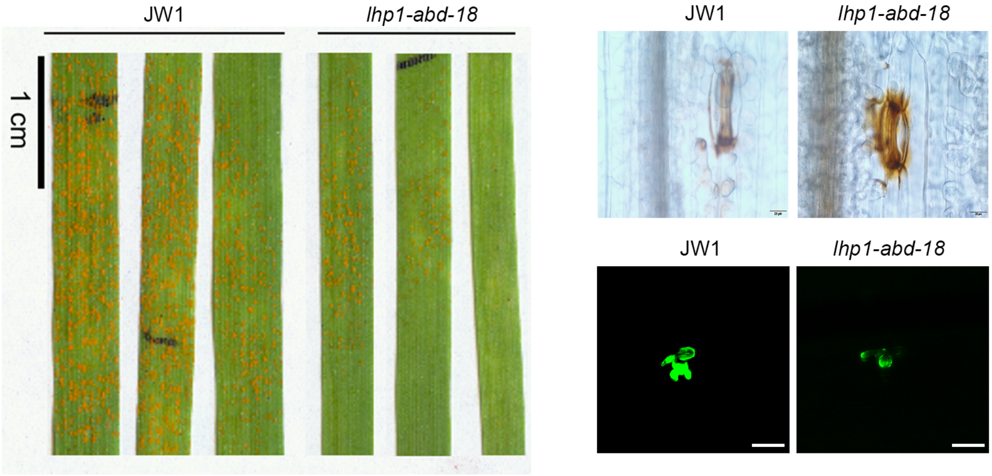

Supplemental Figure 9. Resistance phenotypes to stripe rust in *Talhp1-abd-18*. Left: WT and *Talhp1-abd-18* plants were inoculated with *Pst* CYR32 and photographed at 14 DPI; bar, 1 cm. Right top: H_2O_2 accumulation in WT and *Talhp1-abd-18* leaves at 2 DPI revealed by DAB staining; bar, 20 µm. Right bottom: *Pst* growth in wild-type and *Talhp1-abd-18* plants at 2 dpi (stained with wheat germ agglutinin conjugated to Alexa-488) ; bar, 50 µm.

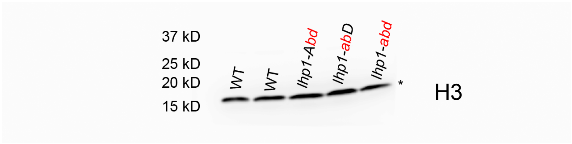

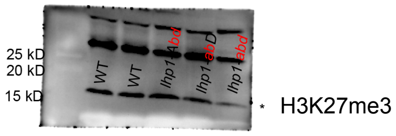

Supplemental Figure 10. The global H3K27me3 level in *Talhp1* triple, single and double mutants via western blot with specific antibody against H3K27me3 (Full uncropped scans of gel).
