# Supplementary material for: LHP1-mediated epigenetic buffering of subgenome diversity and defense responses confers genome plasticity and adaptability in allopolyploid wheat
Source: Nat Commun. 2023 Nov 20;14:7538. doi: 10.1038/s41467-023-43178-2 (PMC10661560; doi:10.1038/s41467-023-43178-2)
Supplement: Supplementary file 3 — Description of Additional Supplementary Files [file 41467_2023_43178_MOESM3_ESM.pdf]

Description of Additional Supplementary Files

Supplemental Data 1. Enrichment and significance of TF targets in sc-triads and non-sc-homoeologs. (TF targets > 10 genes)

Supplemental Data 2. Replicated data in this study.
