# Supplementary material for: LHP1-mediated epigenetic buffering of subgenome diversity and defense responses confers genome plasticity and adaptability in allopolyploid wheat
Source: Nat Commun. 2023 Nov 20;14:7538. doi: 10.1038/s41467-023-43178-2 (PMC10661560; doi:10.1038/s41467-023-43178-2)
Supplement: Supplementary file 6 — Reporting Summary [file 41467_2023_43178_MOESM6_ESM.pdf]

Corresponding author(s): Moussa Benhamed, Zhicheng Dong, Jinying Gou, Yijing Zhang

Last updated by author(s): Oct 8, 2023

Reporting Summary

Nature Portfolio wishes to improve the reproducibility of the work that we publish. This form provides structure for consistency and transparency in reporting. For further information on Nature Portfolio policies, see our Editorial Policies and the Editorial Policy Checklist.

Statistics

For all statistical analyses, confirm that the following items are present in the figure legend, table legend, main text, or Methods section.

n/a	Confirmed	
☐	☒	The exact sample size (*n*) for each experimental group/condition, given as a discrete number and unit of measurement
☐	☒	A statement on whether measurements were taken from distinct samples or whether the same sample was measured repeatedly
☒	☐	The statistical test(s) used AND whether they are one- or two-sided<br>*Only common tests should be described solely by name; describe more complex techniques in the Methods section.*
☒	☐	A description of all covariates tested
☒	☐	A description of any assumptions or corrections, such as tests of normality and adjustment for multiple comparisons
☐	☒	A full description of the statistical parameters including central tendency (e.g. means) or other basic estimates (e.g. regression coefficient) AND variation (e.g. standard deviation) or associated estimates of uncertainty (e.g. confidence intervals)
☒	☐	For null hypothesis testing, the test statistic (e.g. *F*, *t*, *r*) with confidence intervals, effect sizes, degrees of freedom and *P* value noted<br>*Give P values as exact values whenever suitable.*
☒	☐	For Bayesian analysis, information on the choice of priors and Markov chain Monte Carlo settings
☒	☐	For hierarchical and complex designs, identification of the appropriate level for tests and full reporting of outcomes
☒	☐	Estimates of effect sizes (e.g. Cohen's *d*, Pearson's *r*), indicating how they were calculated

Our web collection on statistics for biologists contains articles on many of the points above.

Software and code

Policy information about availability of computer code

Data collection	The libraries of H3K27me3 ChIP-seq data were sequenced with the Illumina NovaSeq 6000 system to produce 150-bp paired-end reads. The libraries of LHP1 ChIP-seq data were sequenced with Hiseq-PE150 to produce 150 bp paired-end reads. The libraries of RNA-seq data were sequenced with the Illumina NovaSeq 6000 system to produce 150-bp paired-end reads. Scripts are available at https://github.com/yuyun-zhang/hexa_LHP1.
Data analysis	fastp (version 0.20.0); Trim Galore (version 0.4.4); Burrows–Wheeler Aligner (version 0.7.17-r1188); HISAT2 (version 2.2.1); MACS (version 2.2.6); featureCount program of the Subread package (version 2.0.0); MAnorm2 (version 1.0.0); mummer (version 4.0.0beta2); MCScanX python version (version 1.0); VCFtools (version 0.1.13); Orthofinder(version 2.3.12); Integrative Genomics Viewer (version 2.8.10)

For manuscripts utilizing custom algorithms or software that are central to the research but not yet described in published literature, software must be made available to editors and reviewers. We strongly encourage code deposition in a community repository (e.g. GitHub). See the Nature Portfolio guidelines for submitting code & software for further information.

Data

Policy information about availability of data

All manuscripts must include a data availability statement. This statement should provide the following information, where applicable:

- Accession codes, unique identifiers, or web links for publicly available datasets
- A description of any restrictions on data availability
- For clinical datasets or third party data, please ensure that the statement adheres to our policy

The ChIP-Seq and RNA-seq data generated in this study have been submitted to the NCBI Gene Expression Omnibus (GEO; https://www.ncbi.nlm.nih.gov/geo/) under accession number GSE218538 (https://www.ncbi.nlm.nih.gov/geo/ GSE218538). Tracks for all sequencing data can be visualized through our local genome browser (http://bioinfo.sibs.ac.cn/LHP1_jbrowse/). The histone ChIP-seq data of Chinese Spring (CS) seedlings used in this study are under accession numbers GSE139019 and GSE121903 in the NCBI GEO database. The H3K27me3 ChIP-seq data for Oryza sativa and Arabidopsis thaliana used in this study were downloaded from the NCBI GEO database (accession numbers GSE67322 and GSE142462). RNA-seq used in Figure 5a were obtained from NCBI SRA database (accession numbers PRJEB12358, PRJEB24686, PRJNA263755, PRJNA289545, PRJNA401295, PRJNA428316, PRJNA450087, PRJNA595999, PRJNA613349, PRJNA630776, PRJNA664832, PRJNA718488, PRJNA749387). The functional genes of T. aestivum were downloaded from WheatOmics(http://wheatomics.sdau.edu.cn/genes/). B. distachyon genomes were obtained from Phytozome (v12). O. sativa from RAP-DB85. Z.mays from MaizeGDB. A. thaliana from TAIR. H. vulgare and T. turgidum from the Plant Genomics and Phenomics Research Data Repository. S. cereale was obtained from the Chinese National Genomics Data Center. T. urartu from MBKBase. A. tauschii from the EnsemblPlants database (Aet_v4.0).

Research involving human participants, their data, or biological material

Policy information about studies with human participants or human data. See also policy information about sex, gender (identity/presentation), and sexual orientation and race, ethnicity and racism.

Reporting on sex and gender	This article does not involve human research.
Reporting on race, ethnicity, or other socially relevant groupings	This article does not involve human research.
Population characteristics	This article does not involve human research.
Recruitment	This article does not involve human research.
Ethics oversight	This article does not involve human research.

Note that full information on the approval of the study protocol must also be provided in the manuscript.

Field-specific reporting

Please select the one below that is the best fit for your research. If you are not sure, read the appropriate sections before making your selection.

☒ Life sciences ☐ Behavioural & social sciences ☐ Ecological, evolutionary & environmental sciences

For a reference copy of the document with all sections, see nature.com/documents/nr-reporting-summary-flat.pdf

Life sciences study design

All studies must disclose on these points even when the disclosure is negative.

Sample size	No statistics method is used to predetermine sample size for RNA-seq and ChIP-seq data. We investigated the expression and epigenetic dynamic in normal and infective condition in wild type and mutant wheat.
Data exclusions	No data was excluded from the analysis.
Replication	We have replicates for all data generated from wild type, including RNA-seq before and after pathogen treatment, H3K27me3 ChIP-seq before and after pathogen inoculation, H3K27me3 ChIP-seq in leaf protoplast, and ChIP-seq of LHP1 binding loci in leaf protoplast (Table S2). We have 2 independent mutant lines. The main results were reproduced in this study (Supplementary Figure 6-9).
Randomization	Because we did not have group allocation in this study, the randomization was not used.
Blinding	Because we did not have group allocation in this study, the blinding was not used.

Reporting for specific materials, systems and methods

We require information from authors about some types of materials, experimental systems and methods used in many studies. Here, indicate whether each material, system or method listed is relevant to your study. If you are not sure if a list item applies to your research, read the appropriate section before selecting a response.

Materials & experimental systems

n/a	Involved in the study
☐	☒ Antibodies
☒	☐ Eukaryotic cell lines
☒	☐ Palaeontology and archaeology
☒	☐ Animals and other organisms
☒	☐ Clinical data
☒	☐ Dual use research of concern
☐	☒ Plants

Methods

n/a	Involved in the study
☐	☒ ChIP-seq
☒	☐ Flow cytometry
☒	☐ MRI-based neuroimaging

Antibodies

Antibodies used: H3K4me3: Abcam, cat #ab8580; H3K9ac: Upstate, cat #07-352; H3K27me3:Upstate-07–449; anti-Flag antibody (A2220, Sigma)

Validation: The antibody has been validated by the supplier. The reports could be found on the the supplier website.

Plants

Seed stocks: We used Common wheat (Triticum aestivum variety JW1) in this study and generated the transgenic lines by our own.

Novel plant genotypes: We generated the transgenic lines by using CRISPR-Cas9. To modify all three TaLHP1 copies, we used two sgRNAs that target conserved regions (target1: AGGTCCTATGGCAAGCGCAA, target2: GAGCAAGCAGCAGGAGAGGT). Synthesized oligos for target-specific sgRNAs were annealed and cloned into the pBUE411 vector. After sequencing of the target sites, the binary vector was transformed into the wheat cultivar JW1 by Agrobacterium tumefaciens-mediated transformation.

Authentication: To determine the editing efficiency, we amplified the two target sites in T0 transgenic plants for Sanger sequencing and found that the first target site was not edited, and the second target site was edited in all three subgenomes. Therefore, in the subsequent genotyping, we used Hi-TOM to detect only the second target sites.

ChIP-seq

Data deposition

☒ Confirm that both raw and final processed data have been deposited in a public database such as GEO.

☒ Confirm that you have deposited or provided access to graph files (e.g. BED files) for the called peaks.

Data access links
May remain private before publication.

The ChIP-Seq and RNA-seq data generated in this study have been submitted to the NCBI Gene Expression Omnibus (GEO; https://www.ncbi.nlm.nih.gov/geo/) under accession number GSE218538 (https://www.ncbi.nlm.nih.gov/geo/query/acc.cgi?acc=GSE218538, reviewer token: efwjeksmjbczvij).

Files in database submission

JW1_Leaf_H3K4me3_rep1.rpm.bw
JW1_Leaf_H3K9ac_rep1.rpm.bw
JW1-H3K27me3-lib.sort.q20.rmdup.rpm.bw
JW1_rep2_H3K27me3.sort.q20.rmdup.rpm.bw
JW1_SR_H3K27me3_v1.sort.q20.rmdup.rpm.bw
JW1.wheat.sort.q20.rmdup.rrpm.bw
L19.wheat.sort.q20.rmdup.rrpm.bw
L18.wheat.sort.q20.rmdup.rrpm.bw
LHP1.sort.q20.rmdup.rpm.bw
JW1-CK-rep2.sort.q20.uniq.rpm.bw
JW1-SR-rep2.sort.q20.uniq.rpm.bw
JW1-pro-K27me3-1.sort.q20.rmdup.rpm.bw
JW1-pro-K27me3-2.sort.q20.rmdup.rpm.bw
LHP1-rep2.sort.q20.rmdup.rpm.bw
JW1_SR_H3K27me3_v2.sort.q20.rmdup.rpm.bw
featurecounts.JW1-CK-rep2.fpkm
featurecounts.JW1-SR-rep2.fpkm
JW1-pro-K27me3-1_PE_peaks.xls
JW1-pro-K27me3-2_PE_peaks.xls
LHP1-rep2_PE_peaks.xls
JW1_SR_H3K27me3_v2_PE_peaks.xls
macs2_JW1_Leaf_H3K4me3_rep1.allgenome.nomodel.nolambda_peaks.xls
macs2_JW1_Leaf_H3K9ac_rep1.allgenome.nomodel.nolambda_peaks.xls
JW1-H3K27me3-lib_PE_peaks.xls

JW1_rep2_H3K27me3_PE_peaks.xls
JW1_SR_H3K27me3_v1_PE_peaks.xls
JW1_PE_peaks.xls
L18_PE_peaks.xls
L19_PE_peaks.xls
LHP1_PE_peaks.xls
JW1_Leaf_H3K4me3_rep1_R1.fastq.gz
JW1_Leaf_H3K9ac_rep1_R1.fastq.gz
JW1-H3K27me3-lib_FKDL202626447-1a_1.fq.gz
JW1_rep2_H3K27me3_1.fq.gz
JW1_SR_H3K27me3_v1_BMRC210003406-1A_1.fq.gz
JW1-at-H3K27me3-lib_BKDL210056190-1a_1.fq.gz
L19-at-H3K27me3-lib_BKDL210056192-1a_1.fq.gz
L18-at-H3K27me3-lib_BKDL210056191-1a_1.fq.gz
LHP1_1.fq.gz
JW1_Leaf_H3K4me3_rep1_R2.fastq.gz
JW1_Leaf_H3K9ac_rep1_R2.fastq.gz
JW1-H3K27me3-lib_FKDL202626447-1a_2.fq.gz
JW1_rep2_H3K27me3_2.fq.gz
JW1_SR_H3K27me3_v1_BMRC210003406-1A_2.fq.gz
JW1-at-H3K27me3-lib_BKDL210056190-1a_2.fq.gz
L19-at-H3K27me3-lib_BKDL210056192-1a_2.fq.gz
L18-at-H3K27me3-lib_BKDL210056191-1a_2.fq.gz
LHP1_2.fq.gz

Genome browser session
(e.g. UCSC)

Tracks for all sequencing data can be visualized through our local genome browser (http://bioinfo.sibs.ac.cn/LHP1_jbrowse/).

Methodology

Replicates

Line lhp1-abd-18 and line lhp1-abd-19 are used to verify the replication of experiment.

Sequencing depth

All ChIP-seq reads were paired-end 150bp. The number of raw read bases were show below:
JW1-H3K27me3-rep1 16323877200
JW1-H3K27me3-spikein 14831527800
JW1-H3K27me3-rep2 30895063800
JW1-H3K27me3-SR 11426997900
L18-H3K27me3-spikein 14892403200
L19-H3K27me3-spikein 20526692100
JW1-LHP1 12563000700
JW1-H3K4me3 12055039800
JW1-H3K9ac 13538327400
JW1-H3K27me3-SR-rep2 9587851200
JW1H3K27me3-protoplast-rep1 11769975000
JW1H3K27me3-protoplast-rep2 10642856400
LHP1-rep2 8446163700

Antibodies

H3K4me3: Abcam, cat #ab8580; H3K9ac: Upstate, cat #07-352; H3K27me3:Upstate-07–449; anti-Flag antibody (A2220, Sigma)

Peak calling parameters

MACS2 was used with parameters: "-f BAMPE -g 14271578887 --nomodel --nolambda" , FDR < 0.05 and P value < 1e-10.

Data quality

The Peaks numbers of the ChIP-seq generated in this article are listed below:
JW1-H3K27me3-rep1 189643
JW1-H3K27me3-spikein 314231
JW1-H3K27me3-rep2 273663
JW1-H3K27me3-SR 123925
L18-H3K27me3-spikein 193897
L19-H3K27me3-spikein 215978
JW1-LHP1 85726
JW1-H3K4me3 164274
JW1-H3K9ac 107068
JW1H3K27me3-protoplast-rep1 7972
JW1H3K27me3-protoplast-rep2 3909
LHP1-rep2 29869
JW1-H3K27me3-SR-rep2 85803

Software

fastp (version 0.20.0); Trim Galore (version 0.4.4); Burrows–Wheeler Aligner (version 0.7.17-r1188); MACS (version 2.2.6); MAnorm2 (version 1.0.0)
